# cnnImpute: missing value recovery for single cell RNA sequencing data

**DOI:** 10.1038/s41598-024-53998-x

**Published:** 2024-02-16

**Authors:** Wenjuan Zhang, Brandon Huckaby, John Talburt, Sherman Weissman, Mary Qu Yang

**Affiliations:** 1 MidSouth Bioinformatics Center and Joint Bioinformatics Graduate Program, University of Arkansas at Little Rock, University of Arkansas for Medical Sciences, Little Rock, 72204 AR USA; 2 Department of Information Science, University of Arkansas at Little Rock, Little Rock, 72204 AR USA; 3 Department of Computer Science, University of Arkansas at Little Rock, Little Rock, 72204 AR USA; 4 Department of Genetics, Yale School of Medicine, New Haven, 06520 CT USA

**Keywords:** Computational biology and bioinformatics, Biomarkers

## Abstract

The advent of single-cell RNA sequencing (scRNA-seq) technology has revolutionized our ability to explore cellular diversity and unravel the complexities of intricate diseases. However, due to the inherently low signal-to-noise ratio and the presence of an excessive number of missing values, scRNA-seq data analysis encounters unique challenges. Here, we present cnnImpute, a novel convolutional neural network (CNN) based method designed to address the issue of missing data in scRNA-seq. Our approach starts by estimating missing probabilities, followed by constructing a CNN-based model to recover expression values with a high likelihood of being missing. Through comprehensive evaluations, cnnImpute demonstrates its effectiveness in accurately imputing missing values while preserving the integrity of cell clusters in scRNA-seq data analysis. It achieved superior performance in various benchmarking experiments. cnnImpute offers an accurate and scalable method for recovering missing values, providing a useful resource for scRNA-seq data analysis.

## Introduction

Single-cell RNA sequencing (scRNA-seq) has rapidly gained prominence over the past few decades as a powerful technique for transcriptome profiling^1^. In contrast to conventional bulk-level RNA sequencing, scRNA-seq measures gene expression at the individual cell level, enabling the dissection of complex tissues and heterogeneous cell populations, the uncovering of hidden layers of cellular diversity, and the identification of previously unknown cell types and states^2–5^. Its applications have extended across a wide spectrum of biological and biomedical fields, from developmental biology^6–9^ to cancer research^10–13^, immunology^14–16^, neuroscience^17–19^, and beyond. With its ability to capture the subtleties of gene expression at the single-cell level, scRNA-seq has enabled the identification of rare cell populations^20^, tracking dynamic cellular changes^21^, and enhancing our understanding of the molecular mechanisms underlying diseases^22^.

However, dropout is a prevalent issue in scRNA-seq data, characterized by the absence of gene expression measurements of certain genes in individual cells. This phenomenon occurs due to a combination of technical and biological factors^23,24^. Technical limitations, such as low RNA content in individual cells and biases during library preparation, can result in the underrepresentation of transcripts in the sequencing data. Biological heterogeneity among cells, where genes are stochastically expressed or selectively active in specific cell states, further contributes to dropout events. Addressing dropout is crucial for accurate analysis and interpretation of scRNA-seq data. Ignoring these missing values can lead to biased downstream analysis and the obscuring of essential biological insights.

To address the challenge of excessive missing values in scRNA-seq data, a range of computational and statistical methods has been developed, including recent imputation techniques such as ALAR (Adaptive Low-rank Autoregressive)^25^, bayNorm^26^, DCA (Deep Count Autoencoder)^27^, DeepImpute^28^, MAGIC^29^, scGNN (Single-cell Graph Neural Network)^30^, scImpute^31^, scVI (Single-cell Variational Inference)^32^, SAVER^33^, and SDImpute (Sequential Denoising Imputation)^34^. ALAR leverages adaptive low-rank autoregressive models, considering temporal dependencies in gene expression profiles. bayNorm, a Bayesian method, employs a hierarchical framework, capturing both global and gene-specific characteristics by borrowing information across genes and cells. DCA, a deep learning-based method, utilizes a deep count autoencoder to capture complex dependencies and handle dropout events. DeepImpute employs a fully connected neural network and utilizes a threshold based on standard deviation to determine missing probability. MAGIC relies on a graph-based approach for missing value recovery, preserving global expression patterns but may smooth data points, making it challenging to distinguish dropout events. SAVER, a Bayesian framework, models the relationship between dropout probabilities and expression levels to estimate missing values. scGNN employs graph neural networks, considering cellular neighborhood relationships. scImpute uses a mixture model to detect dropout events and impute missing values by borrowing information from neighboring cells without dropouts. SDImpute relies on singular value decomposition for imputation. scVI is a generative model-based method, employing a variational autoencoder to learn the underlying distribution of scRNA-seq data.

In recent years, Convolutional Neural Networks (CNNs) have achieved great success in computer vision applications, such as image classification, object detection, and facial recognition, as well as natural language processing tasks, demonstrating their versatility and effectiveness across diverse domains. CNNs excel in learning intricate patterns and extracting hierarchical features from data, motivating us to develop a new CNN-based imputation model to recover missing values in scRNAseq data. Such a model can learn the expression correlation within neighboring genes and effectively discern subtle variations and dependencies through its convolutional layers. In contrast to simple neural networks, which may struggle to capture these intricate spatial dependencies. CNNs demonstrate efficiency in handling large-scale datasets, making them practical for the ever-growing scRNAseq datasets.

Here, we introduce cnnImpute, a novel imputation method based on CNN, designed for missing value recovery. Our approach employs a gamma-normal distribution to estimate dropout probabilities, focusing on imputing only those expression values with a high likelihood of being missing. In contrast to fully connected layers in a standard neural network, our CNN-based approach effectively reduces the high dimensionality of input data without sacrificing information and supports parameter sharing, enhancing efficiency and ease of training. The imputation of missing gene values is based on their co-expressed genes. Comprehensive assessments on both real scRNA-seq and simulated datasets, utilizing various evaluation metrics, demonstrate that cnnImpute provides a reliable and robust imputation method, achieving superior performance compared to existing approaches.

## Results

### Overview of cnnImpute

**Figure 1:**
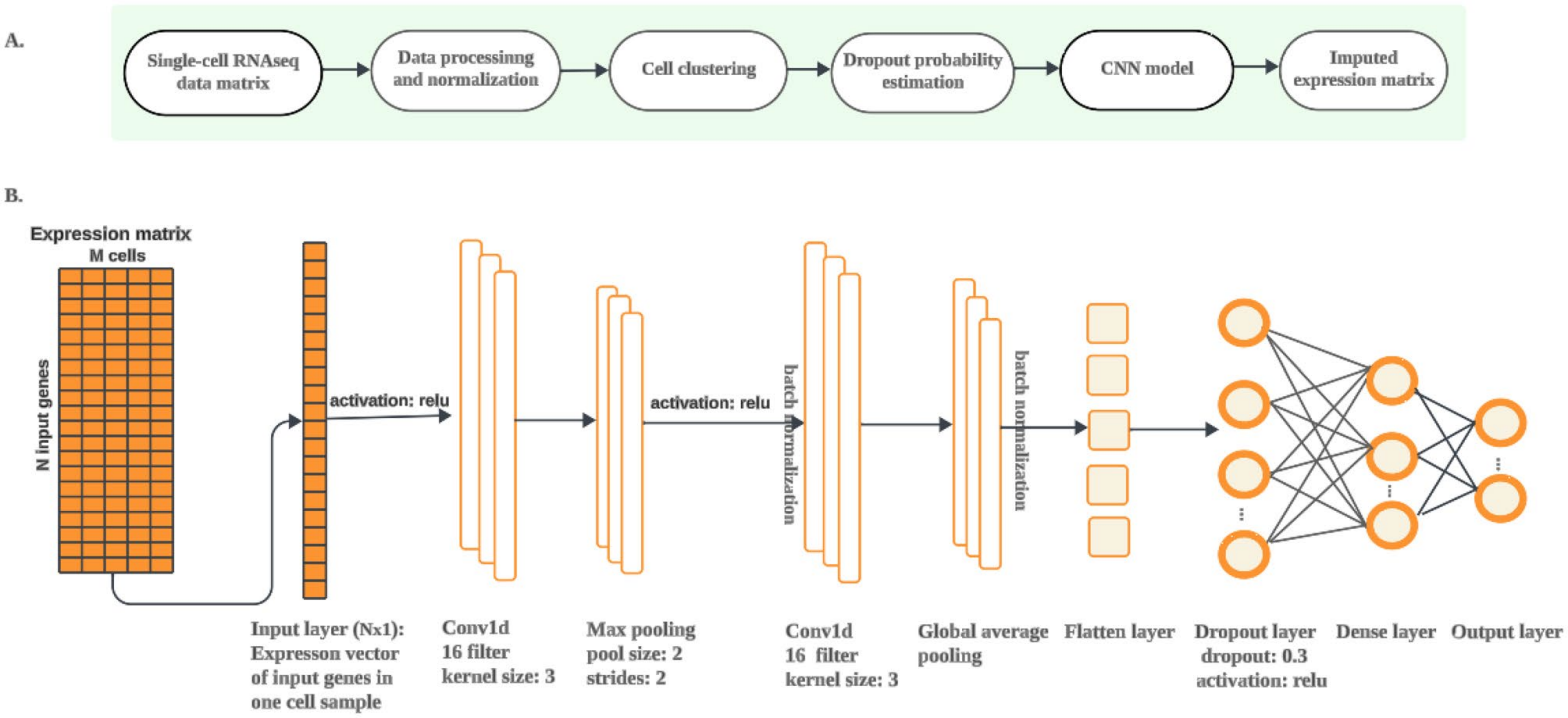
(**A**) The cnnImpute procedure consists of three main steps: data preprocessing, calculation of missing probabilities, and data imputation. (**B**) The architecture of the 1D convolutional neural network-based data imputation model is employed in cnnImpute. The input layer of the CNN model comprises a vector (N $\times$ 1) representing the expression of N = 2560 input genes in a cell, predicting the expression of 512 target genes within the same cell. The expression data (N $\times$ M) of N input genes in M cells is fed into the CNN architecture cell-by-cell with a batch size of 32.

The main steps of cnnImpute include data preprocessing, missing probability assessment, and missing value recovery using a convolutional neural network-based model (Fig. 1A). First, cells with no expressed genes and genes that are not expressed in any of the cells are removed. Subsequently, the remaining data undergoes dimensional reduction using t-SNE, followed by clustering using ADPclust and k-means^35^, resulting in the formation of M cell clusters. An expectation-maximization (EM) algorithm with a gamma-normal distribution^31^ is employed to compute the dropout probability of expression values (Methods). Values with a probability exceeding a threshold T are considered missing, with 0.5 as the default threshold. When the expression of a gene has a dropout probability surpassing the threshold T in any cell, the gene is treated as a target for imputation. The missing values of the target genes are recovered using genes whose expression levels are highly correlated with the target gene. These highly correlated genes are utilized as input for the CNN model. The target genes are divided into subsets, each containing N genes, with 512 as the default value for N (Methods). An individual CNN model is constructed for each target gene subset, enhancing performance robustness and expediting the training process. The architecture of the CNN model is illustrated in Fig. 1B. Further details of the network hyperparameters, training, and testing are provided in the Methods section.

### cnnImpute accurately recovers missing value

We examined the accuracy of missing value recovery using cnnImpute on two real scRNA-seq datasets. The first dataset was generated from human Jurkat cells^5^, while the second dataset was obtained from a mouse study by Grun et al.^36^. For the accuracy assessment, we initially extracted the expression profiles of genes expressed in at least half of the cells. Subsequently, we randomly masked ten percent of the non-zero data points in the selected expression matrix. By varying the random seed for generating masked data points, we tested four additional datasets to evaluate the robustness of performance. After the missing value recovery, we calculated the mean square error (MSE) and Pearson correlation coefficients (PCCs) based on the predicted expression by the imputation method and the true expression levels of the masked-out data points for accuracy assessment. We compared cnnImpute’s performance against other 10 imputation approaches, including ALRA, bayNorm, DCA, DeepImpute, MAGIC, SAVER, scGNN,scImpute, scVI and SDImpute.

**Figure 2:**
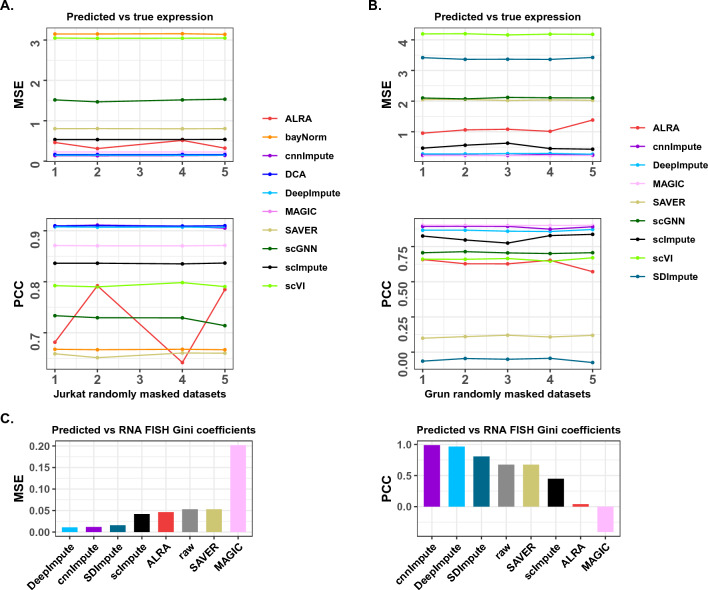
Accuracy evaluation of imputation methods. (**A**) Comparison of mean squared error (MSE) and Pearson correlation coefficient (PCC) values, calculated using predicted expressions and randomly masked-out true expression values for the Jurkat dataset. (**B**) Comparison of MSE and PCC for the Grun dataset. The random mask of non-zero expression values was implemented multiple times with different random seeds to assess performance robustness. (**C**) The MSE and PCC values of of GINI coefficients for genes in RNA FISH data and their corresponding scRNA-seq data, indicating the similarity in gene expression distribution across samples.

cnnImpute consistently exhibited superior performance, achieving the highest Pearson correlation coefficient (PCC) (*P* < 0.014) with the true expression values and the lowest mean square error (MSE) (P < 0.014) in all five randomly masked-out Jurkat datasets, closely followed by DeepImpute and DCA (Fig. 2A, Supp. Fig. 1A showing the top-performing methods). The significance of the performance difference across the five randomly masked datasets of cnnImpute against other imputation methods was assessed using the Wilcoxon test. For the other imputation methods, the MSE-based ranks were MAGIC, ALAR, scImpute, SAVER, scGNN, scVI, and bayNorm, while the PCC-based ranks were MAGIC, scImpute, scVI, scGNN, ALRA, bayNorm, and SAVER (Fig. 2A, Supp. Fig. 1A showing the top-performing methods). SDImpute failed in the Jurkat dataset, whereas both bayNorm and DCA failed in the Grun dataset. For five randomly masked-out datasets from Grun, despite MAGIC exhibiting slightly better performance than cnnImpute (Fig. 2B, Supp. Fig. 1B showing the top-performing methods, pink line vs purple line), cnnImpute outperformed other methods in both MSE and PCC measurements (P < 0.0039). For the remaining imputation methods, the MSE-based ranks were DeepImpute, scImpute, ALRA, SAVER, scGNN, SDImpute, and scVI, while the PCC-based ranks were DeepImpute, scImpute, scGNN, scVI, ALRA, SAVER, and SDImpute (Fig. 2B, Supp. Fig. 1B showing the top-performing methods).

To further evaluate imputation accuracy, we employed an additional method that compared gene expression distributions between single-cellRNA FISH directly measures RNA transcripts in individual cells. In this evaluation, we used RNA FISH data alongside scRNA-seq data (GSE99330) generated from a melanoma cell line. We utilized GINI coefficients to quantify the gene expression distribution. Initially, outlier cells were removed, and subsequent normalization of single-cell and RNA FISH data utilized the GAPDH housekeeping gene. Employing the ADPclust method, we clustered the data and selected the cluster with the highest number of variant genes for further analysis. In alignment with DeepImpute, we retained five genes with a variance-to-mean ratio exceeding 0.5 and present in both the RNA FISH and scRNA-seq datasets. Gini coefficients for these genes were computed across RNA FISH, raw scRNA-seq, and imputed scRNA-seq data (Supp. Fig. 2). Subsequently, we calculated the mean square error (MSE) and Pearson correlation of Gini coefficients between RNA FISH and both raw and imputed scRNA-seq data. bayNorm, DCA, scGNN, and scVI failed in this dataset. Among the imputation approaches that successfully generated imputed data, cnnImpute exhibited the highest correlation value and the second-lowest MSE; its MSE was slightly higher than DeepImpute (Fig. 2C). For the remaining imputation methods, the MSE-based ranks were SDImpute, scImpute, ALRA, SAVER, and MAGIC, while the PCC-based ranks were SDImpute, SAVER, scImpute, ALRA, and MAGIC (Fig. 2C). While MAGIC performed well in random masked-out experiments, it showed poor performance in RNA FISH data assessment compared to other methods. When comparing results with those obtained from the raw scRNA-seq data, the correlation of Gini coefficients between RNA FISH and imputed scRNA-seq data using cnnImpute showed a significant increase from 0.78 to 0.989, while the MSE decreased from 0.033 to 0.012. Collectively, cnnImpute consistently demonstrated reliable accuracy across a variety of datasets.

### cnnImpute Improves cell type detection

Single-cell sequencing enables the dissection of cellular diversity and the discovery of new cell types. However, the presence of missing values in the data often introduces inaccuracies in cell clustering analysis, necessitating the recovery of these missing values. To conduct the evaluation, we applied cnnImpute, cnnImpute_nl, and 10 other existing imputation methods as stated in the previous section, respectively, for missing value recovery. cnnImpute_nl is a variant of cnnImpute that uses expression data without log transformation for model construction.

**Figure 3:**
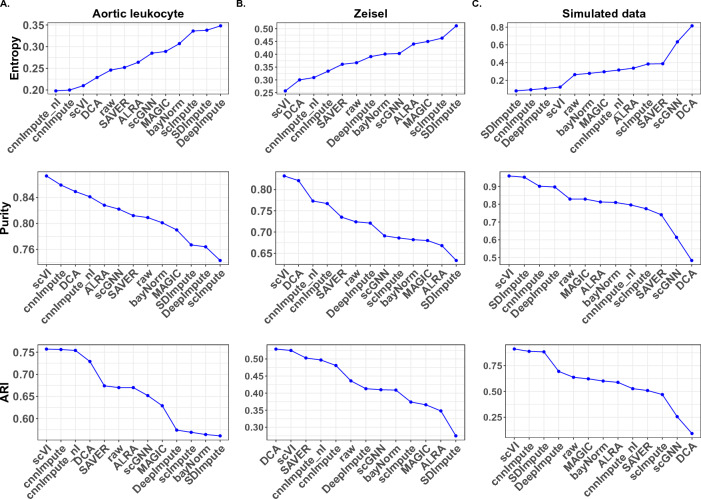
Evaluations of the quality of cell clusters inferred from raw and imputed data using different methods.

Subsequently, cell clusters were inferred from both the raw data and the imputed datasets using ADP clustering in conjunction with k-means. The quality of the resulting cell clusters was assessed using metrics, including purity, entropy, and the Adjusted Rand Inde $\times$ (ARI) (Methods). Entropy is a measure of impurity within a cluster, where 0 indicates perfect purity, and higher values, approaching 1, indicate impurity. Purity falls within a range from 0 to 1; a higher purity implies that cells in each cluster are more accurately and distinctly grouped with minimal mixing between clusters. The ARI quantifies the similarity between predicted clustering and true clustering, with a higher ARI score suggesting that the predicted clustering successfully partitions the cells into clusters closely resembling the true cell clusters.

Three datasets were used in the evaluation: two real single-cell RNA sequencing datasets, namely mouse aortic leukocyte from PanloaDB with eight distinct immune cell types and the Zeisel dataset from GEO with seven cell types, along with a simulated dataset generated using Splatter. The simulated dataset initially comprised five distinct cell types with varying counts (199, 197, 346, 427, and 831). A dropout mechanism was applied to simulate missing values (see ’Methods’ for details). Before analysis, we filtered the datasets by removing genes not expressed in any cells and eliminating cells with no expressed genes.

In the entropy assessment, cnnImpute and cnnImpute_nl claimed the first position in the aortic leukocyte dataset, with cnnImpute securing the third spot after scVI and DCA in the Zeisel dataset and the second spot following SDImpute in the simulated dataset. While DCA secured the second position in the Zeisel dataset, it exhibited the highest entropy in the simulated dataset. SDImpute demonstrated the smallest entropy in the simulated data; however, it had the largest entropy in the Zeisel data and the second-highest entropy in the aortic leukocyte data. Similarly, DeepImpute claimed the third spot in the simulated dataset; however, it exhibited the highest entropy in the aortic leukocyte data and higher entropy than the raw Zeisel data. In contrast, cnnImpute demonstrated stable superior entropy and improved entropy in the raw data in all three datasets (Fig. 3) top panel).

In terms of purity measurement, cnnImpute ranks second, following scVI, in the aortic leukocyte dataset, while both cnnImpute_nl and cnnImpute rank third and fourth, respectively, following scVI and DCA in the Zeisel dataset. In the simulated dataset, cnnImpute secures the third position, following scVI and SDImpute. Despite SDImpute having higher purity than cnnImpute, it exhibited the smallest purity value in the Zeisel dataset, the third-largest purity in aortic leukocyte, and, additionally, higher purity than the raw data in both real datasets. On the other hand, DCA showed the smallest purity in the simulated dataset. Nevertheless, cnnImpute consistently showed stable purity measurements across all three datasets (Fig. 3) middle panel).

In the ARI assessment, cnnImpute ranked second following scVI, and cnnImpute_nl in the aortic leukocyte dataset, while cnnImpute_nl and cnnImpute secured the fourth and fifth positions in the Zeisel dataset, and the second position in the simulation dataset. Despite DCA having a higher ARI value in the Zeisel data than cnnImpute, it exhibited the lowest ARI in the aortic leukocyte dataset and the simulated data. Similarly, SAVER had a higher ARI than cnnImpute; however, it showed a lower ARI in the aortic leukocyte and simulated datasets (Fig. 3, bottom panel).

**Figure 4:**
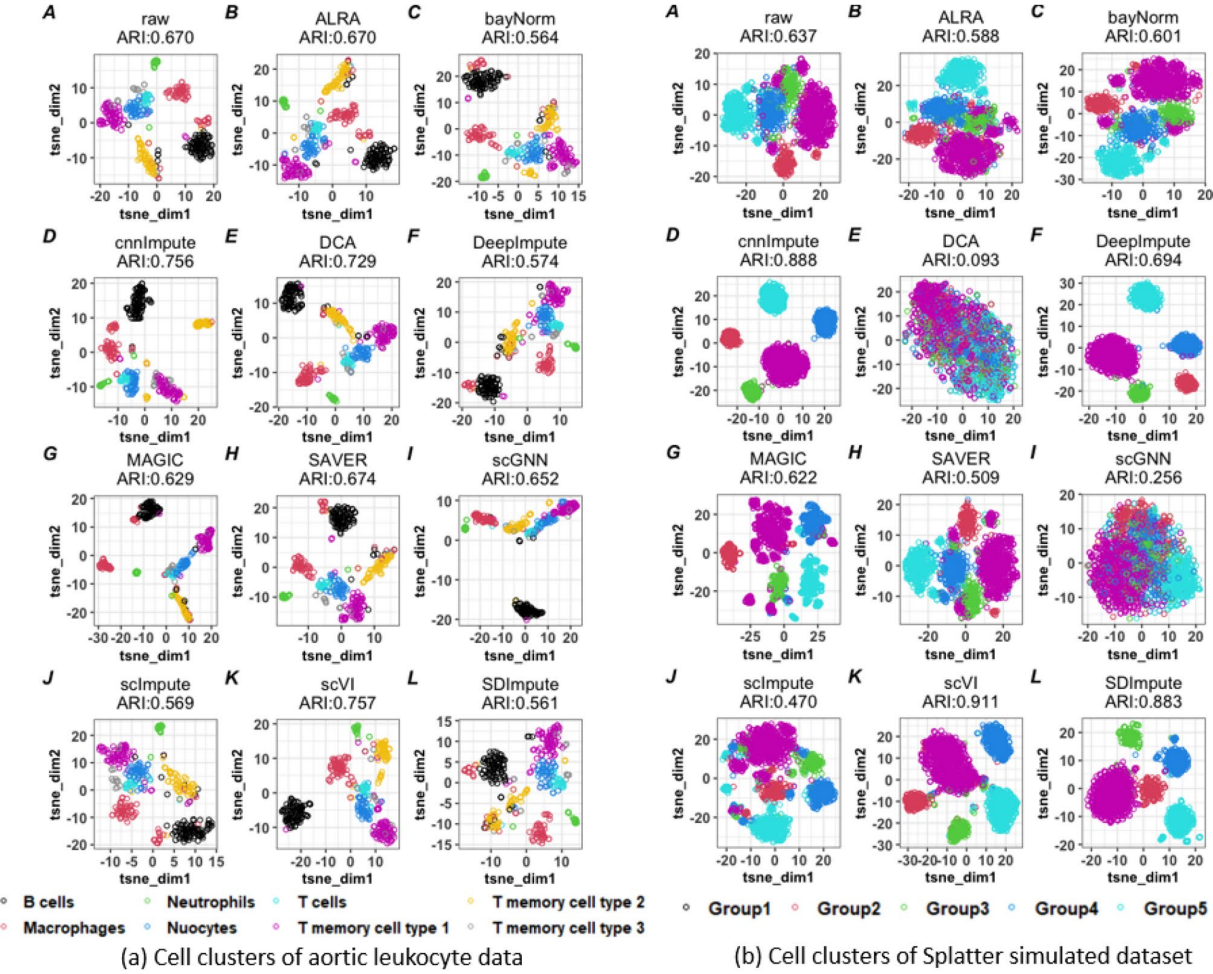
The t-SNE plots of cell clusters from raw scRNAseq data and the corresponding imputed data using various methods for aortic leukocyte and simulated datasets.

Importantly, the imputed data generated by cnnImpute improved the quality of cell clustering compared to the clusters formed using the raw data, as indicated by enhancements in purity, entropy, and ARI scores across all three datasets (Fig. 3), suggesting its effectiveness in improving cell type identification. Collectively, our results suggested that scVI and cnnImpute are the two most stable imputation methods for improving cell cluster detection. These enhanced clustering results are further substantiated by the tSNE plot (Fig. 4).

### cnnImpute enhances on differential expression analysis

**Figure 5:**
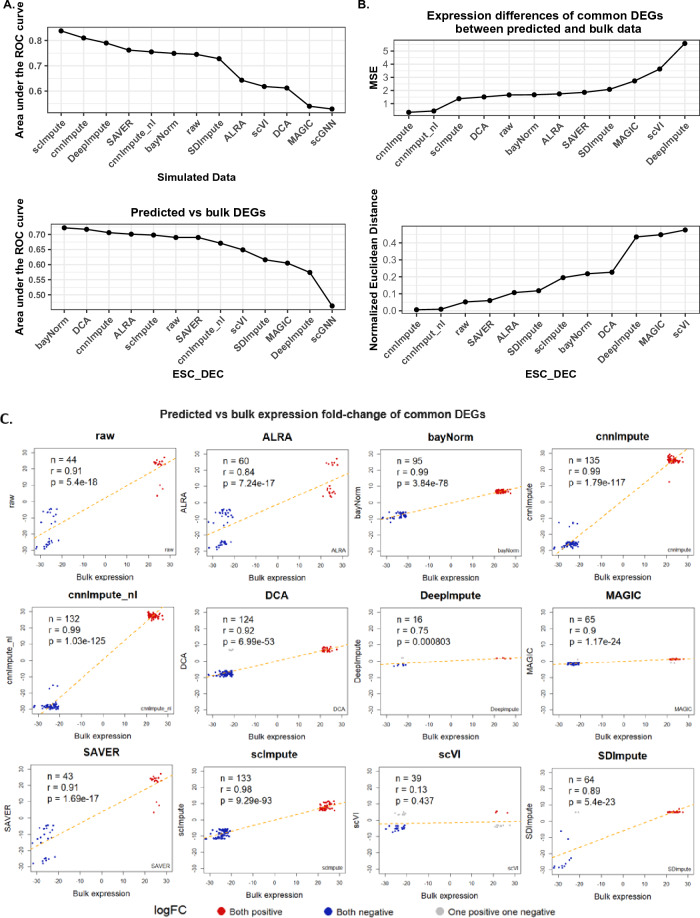
Evaluation of the impact of missing value recovery on differential expression analysis. A. AUC comparison based on reference DEGs and predicted DEGs derived from raw and imputed scRNAseq data using various methods. For ESC-DEC data, reference DEGs were obtained from bulk RNAseq data, while for simulated data, reference DEGs were derived from data before introducing missing values. B. Assessment of fold-change similarity in these common genes using MSE and normalized Euclidean distance. DEGs inferred from imputed data by cnnImpute showed patterns most similar to the reference DEGs. C. Correlation of log-fold change in common genes between the top 300 DEGs from bulk RNAseq data and the top 900 DEGs identified in the raw data and from each imputed dataset, respectively, in the ESC-DEC data. cnnImpute identified the highest number of common DEGs with the strongest fold-change correlation with bulk DEGs.

Differential expression analysis is a fundamental downstream analysis of RNA sequencing data. It is essential for detecting gene signatures related to various aspects, such as cell types and cell lineage development, and it plays a critical role in studying signaling pathways and networks involved in various biological processes. We initially conducted comparisons using a simulated dataset generated by Splatter, which was the same dataset used in the cell clustering analysis. Scanpy was employed for differential expression analysis. The differentially expressed genes (DEGs) identified using the raw data before the introduction of the dropout mechanism for simulating missing values were considered the true DEG set (Methods). To assess the effectiveness of imputed datasets in identifying DEGs, we used the area under the curve (AUC), which measures a range of sensitivity and specificity. Both scImpute and cnnImpute outperformed other methods, followed by DeepImpute, SAVER, cnnImpute_nl, bayNorm, SDImpute, ALRA, scVI, MAGIC, and scGNN, as indicated by AUC values (Fig. 5A top panel). Although cnnImpute ranked second in the simulated dataset (AUC 0.81 vs. 0.838), it still significantly improved the AUC values of the raw dataset, increasing them from 0.745 to 0.81 (Fig. 5A top panel).

Next, we conducted an analysis and comparisons using a real single-cell RNA sequencing (scRNAseq) dataset. In scenarios where scRNAseq profiles the expression of cells with low-level cell-cell heterogeneity within the same cell type, we anticipated that the differentially expressed genes (DEGs) identified from single-cell sequencing would match those found in bulk-level RNA sequencing analyses within the same phenotype pair comparison. Hence, we utilized paired bulk and single-cell RNAseq datasets of human embryonic stem cells (ESC) and definitive endoderm cells (DEC) from GSE75748. The bulk RNAseq data was generated from four H1 ESC samples and two DEC samples, while the scRNAseq data was generated from 212 H1 ESCs and 138 DECs. Notably, the bulk RNAseq dataset exhibited a substantially lower percentage of zeros compared to the original scRNAseq dataset, with values of 14.8% and 49.1%, respectively. We identified DEGs using Scanpy for both the raw scRNAseq dataset and the imputation methods(Methods). These DEGs were ranked in descending order based on their absolute logFC values. To assess performance, we used DEGs identified through bulk RNAseq as the reference and calculated the AUC. Following bayNorm and DCA, the DEGs identified in the imputed scRNAseq data by cnnImpute exhibited the third-largest AUC, succeeded by ALRA, scImpute, SAVER, cnnImpute_nl, scVI, SDImpute, MAGIC, DeepImpute, and scGNN (refer to Fig. 5A bottom panel)

We also examined the expression correlation of concordant DEGs in both bulk scRNAseq datasets (top 300 DEGs) and scRNAseq datasets (top 900 DEGs). In these comparisons, cnnImpute consistently ranked first. Notably, when compared to the raw scRNAseq data, cnnImpute significantly improved the expression correlation coefficient from 0.91 to 0.99 with the bulk dataset. The number of overlapping up-regulated genes with the bulk increased from 16 to 73, while down-regulated genes increased from 26 to 62 (Fig. 5C). Furthermore, cnnImpute achieved the highest overlap rates and expression correlation with the bulk dataset followed by bayNorm, scImpute,DCA, SAVER, MAGIC, SDimpute, ALRA and DeepImpute, scVI (Fig. 5C). Additionally, DEGs imputed by cnnImpute exhibited the most similar expression levels to those of DEGs in the bulk dataset, as measured by both Mean Square Error (MSE) when bulk-level expression levels were used as a reference and normalized Euclidean distance (Fig. 5B). These assessment results underscore the effectiveness of cnnImpute in improving downstream differential expression analysis.

### Comprehensive performance evaluation

**Figure 6:**
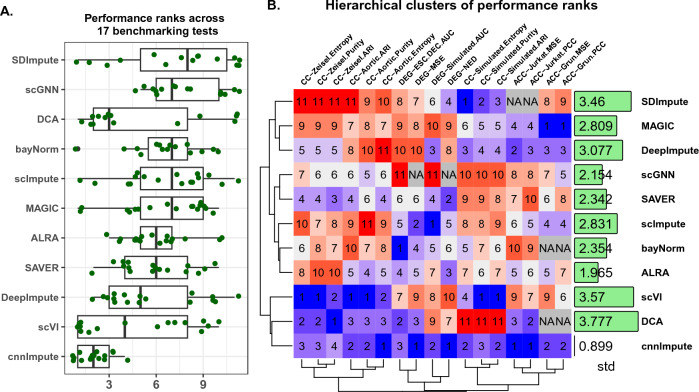
(**A**) The performance rankings of cnnImpute and ten other imputation methods in 17 experiments for assessing accuracy, cell clustering, and the detection of differentially expressed genes (DEG). (**B**) The hierarchical clustering is based on the performance ranks of various imputation methods, with ACC prefix denoting accuracy testing, CC prefix representing cell type identification, and DEG prefix representing the detection of differentially expressed genes. The numbers in the green box represent the standard deviation of performance ranks across various experiments.

In the performance assessment across 17 experiments, which evaluated cnnImpute and compared it against other existing imputation methods, cnnImpute consistently demonstrated superior stability and performance, as depicted in Fig. 6, securing the top rank in diverse assessments across different datasets.

scVI appeared to secure the No.1 rank in cell clustering testing, followed by cnnImpute. However, the performance rank of scVI substantially declined in accuracy and DEG tests with a performance rank variation of 3.57 (Fig. 6B), in contrast to the more stable performance of cnnImpute (std=0.899). DCA exhibited consistent performance in various real scRNAseq datasets, securing the second position overall, but experienced failures in some tests and performed poorly in simulation datasets, resulting in a performance variation of 3.777.MAGIC excelled in accuracy testing in the Grun dataset but performed inadequately in cell type and DEG identification. scImpute ranked high in DEG analysis but lagged in accuracy and cell clustering experiments. On the other hand, SDimpute performed well solely in simulation data. Overall, cnnImpute emerged as the top performer in the comprehensive evaluations with the least performance variation of 0.899 (Fig. 6A,B).

## Discussion

In this study, we introduced cnnImpute, a novel imputation approach that employs a 1D convolutional neural network to recover missing values in scRNA-seq datasets. The designed model architecture facilitates the learning of hierarchical features across neighboring genes through convolutional layers, capturing contextual information embedded in gene expression sequences. Its adaptive adjustment to varying local patterns and proficiency in automatically learning relevant features enable the discernment and recovery of missing values in a data-driven manner. In cnnImpute, the input layer of the CNN consists of genes arranged sequentially based on expression correlation to the targets; the convolutional layers can effectively detect local patterns of input genes relative to the corresponding target genes. In contrast, the order of cells in a cluster is often randomly arranged in the raw data, making a 1D model a more suitable choice. Additionally, a 1D CNN model offers advantages, including robust performance with limited data, lower computational complexity compared to other deep learning architectures and 2D CNNs, a faster training process, and an effective ability to extract relevant features from sequential data. A 1D CNN generally requires fewer parameters, leading to improved computational efficiency. Taken together, cnnImpute leverages the power of a convolutional neural network with the characteristics of scRNA-seq data, offering a unique and promising missing value recovery method.

Given the abundant dropout events in scRNA-seq data, accurate imputation plays a pivotal role in preventing downstream analysis from being compromised by missing data. Our approach was designed to address key challenges in imputation, including selectively recovering actual missing values while preserving true low-expression values and harnessing the intrinsic data structure for missing value recovery. When compared to ten state-of-the-art scRNA-seq imputation methods, including ALRA, bayNorm, DCA, DeepImpute, MAGIC, SAVER, scGNN,scImpute, scVI and SDImpute, cnnImpute consistently demonstrated superior and robust performance across various types of comparisons and validations, utilizing both real and simulated datasets.

The imputation methods evaluated in this study demonstrated varying strengths and weaknesses. ALRA can adaptively smooth data, enhancing its robustness to local fluctuations, and it performs well in scenarios where data exhibit local patterns and varying levels of noise. However, it may encounter challenges when dealing with highly nonlinear relationships and complex datasets. ALRA demonstrated moderate performance compared to other methods, with cnnImpute surpassing it in all 17 tests (Fig. 6A,B). On the other hand, bayNorm utilizes a Bayesian framework, excelling in capturing variability and uncertainty. However, its performance can be influenced by the choice of prior distributions, and it may become computationally expensive for large-size data, potentially affecting its efficacy in large-scale applications. While bayNorm achieved the highest AUC value in DEG identification for ESC-DEC data compared to other methods, it was outperformed by cnnImpute in the remaining 16 benchmarking tests. DCA is designed to model dropout events in scRNA-seq data, allowing it to recap complex dependencies in the data. However, its performance may be affected by outliers. While DCA performed well in real scRNA-seq datasets, it showed comparatively subpar performance in simulated datasets. In contrast, SDImpute , which employs a sequential denoising strategy, excelled in handling missing data in simulation scenarios but demonstrated lower performance in real datasets. scGNN , leveraging graph neural networks, captured intricate relationships in single-cell RNA-seq data. However, its success depended heavily on the quality of underlying graph construction, exhibiting moderate performance in various tests and being outperformed by cnnImpute in all 17 benchmarking experiments. scVI utilizes a variational autoencoder (VAE) framework, combining probabilistic modeling with deep learning techniques, to capture the inherent complexity of single-cell expression profiles. However, training scVI can be computationally demanding; it appeared to be the slowest method suggested by our results. It performed well in cell cluster evaluation tests but underperformed in accuracy assessment and DEG identification according to our results.

Notably, unlike MAGIC, which has a tendency to inflate zeros within scRNA-seq data, potentially obscuring biologically significant variability, cnnImpute excels in selectively recovering the transcripts with a high probability of being missing, allowing it to retain biological variability. While DeepImpute did not recover all zero values in the matrix, it relied solely on the standard deviation to assess the likelihood of a zero value being missing, without fully considering the global distribution of data. Additionally, DeepImpute imputes genes using all cell samples, but gene expression can be cell-type-specific. In other words, a co-expressed gene set varies in different cell types. Hence, DeepImpute’s design may not handle imputation effectively under these circumstances. Consequently, the performance of MAGIC and DeepImpute is relatively low compared to other imputation methods. cnnImpute outperforms MAGIC and DeepImpute significantly in benchmark experiments of differential expression analysis. SAVER is a Bayesian-designed model that requires significant computational resources and time; consequently, it may not scale well to very large datasets. One of the key assumptions underlying SAVER is the modeling of the relationship between dropout probabilities and expression levels, in which genes with low true expression levels are more likely to experience dropout events, while highly expressed genes are less likely to drop out. This may explain the poor performance of SAVER in the accuracy assessment, which masked out the non-zero values and then computed MSE and PCC measurements based on the predicted and true expression values. In comparison to scImpute, which builds a regression model for each cell and subsequently imputes the missing value of a gene using expression levels of this gene in neighboring cells, cnnImpute leverages a deep learning model to predict missing values of a gene based on expression levels of its expression-correlated genes in similar cells. A high missing rate in a gene can adversely impact the precision of missing value prediction under the strategy adopted by scImpute, while cnnImpute effectively harnesses information from co-expressed genes, thereby mitigating the impact of the high missing rate. This advantage is evident by the MSE and enhanced Pearson correlation coefficient PCC achieved by cnnImpute compared to scImpute when comparing the imputed data against the true expression in multiple datasets. Additionally, cnnImpute offers the flexibility to partition data into subnetworks, enhancing its potential scalability for handling large datasets. In summary, cnnImpute provides a stable and accurate missing value recovery approach for facilitating single-cell-based research.

We also introduced a variant of cnnImpute called cnnImpute_nl, which operates on raw data without log transformation. The performance of cnnImpute_nl showed similar clustering quality to cnnImpute, but it exhibited lower performance in DEG identification. This difference in performance may be attributed to outliers in the raw data or the dominating effect of highly expressed genes, such as housekeeping genes. Consequently, subtle differences among genes may be overlooked. As most existing methods operate on log-transformed data, fairly comparing cnnImpute_nl with other imputation methods is not straightforward. Nevertheless, cnnImpute_nl still outperforms several methods that use log-transformed data in various evaluations.

In the benchmarking experiments for running speed, cnnImpute was faster than scImpute and scVI but slower than ALAR, DCA, DeepImpute, bayNorm, and SAVER (Supp. Fig. 4). More than half of the computation time for cnnImpute is dedicated to the evaluation of missing probabilities. Skipping this step makes the speed of cnnImpute comparable to that of DeepImpute. In the peak memory comparison, the three deep-learning-based methods-DCA, cnnImpute, and DeepImpute-had larger memory usage than other imputation methods, namely scVI, bayNorm, scImpute, SAVER, ALAR, and SDImpute (Supp. Fig. 4). Nevertheless, our method strikes a good balance between speed, memory usage, and accuracy, consistently achieving robust results across a variety of tasks.

cnnImpute adopted gamma and normal distributions for estimating dropout probability. While a number of studies have shown that the mixture model of gamma-normal distribution closely approximates gene expression in scRNA-seq datasets, the estimation may still introduce false positives and false negatives, similar to any other distribution models, owing to complexities in biological data. However, through extensive evaluations in this study, the reliable and robust performance of cnnImpute, especially when contrasted with other methods that lack systematic evaluation of dropout events or simply define the dropout event, such as MAGIC and DeepImpute, indicates that this model is suitable for generalizing dropout probabilities in scRNA-seq data. The missing values of target genes were recovered using input genes. For a dataset with very high sparsity, the limited availability of input genes correlated with target genes may impact the model’s performance. Nevertheless, an scRNA-seq dataset with very high sparsity poses a challenge for any imputation method. Conversely, an excessively sparse scRNA-seq dataset may indicate the need for improvements in data generation to ensure reliable biological discovery. Despite potential limitations, cnnImpute’s consistent and superior performance suggests its value for scRNA-seq data analysis. Furthermore, its capacity for pre-training and compatibility with rapidly advancing hardware infrastructure positions cnnImpute as a promising approach capable of keeping pace with the ever-growing size of scRNA-seq data.

## Conclusions

In this work, we developed cnnImpute, a convolutional neural network-based method, for recovering missing data in scRNA-seq datasets. Through extensive assessments and comparisons with other state-of-the-art approaches using multiple real and scRNA-seq datasets, our method consistently outperformed the existing methods. These findings demonstrate the effectiveness of cnnImpute and its potential as a valuable solution for data imputation. Our work establishes a novel and accurate data imputation method for enhancing scRNA-seq data analysis.

## Methods

### Data preprocessing and dropout probability calculation

After filtering out low-quality cells and unexpressed genes across all cells, we proceeded with library normalization, ensuring that each library contained a total of $10^6$ reads. Subsequently, we applied a logarithm transformation to the expression values. To prevent infinite values resulting from zeros, we introduced a correction by adding one to individual expressions before the logarithmic transformation. To perform dimensionality reduction, we employed the R function Rtsne to implement t-distributed stochastic neighbor embedding (t-SNE) with a seed value of 1. The parameters for the function were configured with $dims = 3$, while default values were retained for other parameters. For determining the optimal number of clusters, we utilized the adpclust function from the R package ADPclust, which offers an automatic centroid calculation. The ADP clustering method employs adaptive density peak detection utilizing nonparametric multivariate kernel estimation^35^ This approach facilitates a fast search for cluster centers, accomplishing results in a single step without the need for iteration. It holds great potential for efficient large-scale data analysis. Subsequently, we applied k-means clustering to label the data based on the number of clusters and the corresponding centroids estimated by ADPclust.

We employed a mixture model of gamma and normal distributions to represent actual gene expression and dropout distributions^31,33,37^. The density function of the expression of the $i^{th}$ gene in a cell cluster (*c*) is described by the following equation:

1 $$\begin{aligned} f_{g_i}(X) = \eta _i^c \Gamma (X, \alpha _i^c, \beta _i^c) + (1 - \eta _i^c) \mathscr {N}(X, \mu _i^c, \sigma _i^c) \end{aligned}$$

Here, the dropout is modeled by the gamma distribution ($\Gamma$), while the log-transformed actual gene expression level is modeled by the normal distribution. The symbol $\eta _i^c$ refers to the dropout rate of the $i^{th}$ gene in the cell cluster *c*. The expectation-maximization (EM) algorithm was adapted to estimate the parameters of the gamma-normal model. The resulting estimated parameters ($\hat{\eta }_i^c$, $\hat{\alpha }_i^c$, $\hat{\beta }_i^c$,$\hat{\mu }_i^c$, $\hat{\sigma }_i^c$) were substituted into the following equation to calculate the dropout probability of the $i^{th}$ gene in the $j^{th}$ cell ($\text {DP}_{i,j}$).

2 $$\begin{aligned} \text {{DP}}_{i,j} = \frac{\hat{\eta }_i^c \Gamma (x_{i,j}, \hat{\alpha }_i^c, \hat{\beta }_i^c)}{\hat{\eta }_i^c \Gamma (x_{i,j}, \hat{\alpha }_i^c, \hat{\beta }_i^c) + (1 - \hat{\eta }_i^c) \mathscr {N}(x_{i,j}, \hat{\mu }_i^c, \hat{\sigma }_i^c)} \end{aligned}$$

### CNN-based model construction

After evaluating dropout probabilities, we constructed a CNN based on a deep learning model for imputation. An expression value with a dropout probability exceeding a threshold T, with the default value set to 0.5, is considered missing. Any gene with any missing expression values is regarded as a target gene for imputation. We divided the targeted genes into small subsets, each containing N genes, with the default value of N set to 512. In cases where the size of the last target genes is below 512, we employed padding with -1 for the target genes and padding with a value of 0 for the input genes. This strategy ensures that the introduced padding does not influence the network. The decision to use -1 for padding in target genes is intentional, as all values equal to -1 will be masked during the computation of both the loss and correlation coefficient. The target genes were not sorted in a particular order to avoid potential overfitting. The empirical evidence suggests that the target gene size of 512 results in a good balance between performance and running time (Supp. Fig. 3). A CNN model was trained for each target gene subset.

The input layer of the model comprised genes whose expression levels were highly correlated with the target genes. This was followed by a convolutional layer with a filter size of 16 and a second convolutional layer with 16 filters. Both filters have a kernel size of 3. Both convolutional layers utilized the rectified linear unit (ReLU) activation function and a max-pooling layer. To mitigate overfitting, we introduced a dropout layer with a rate of 0.3. After the dropout layer, there was one dense layer with 1024 nodes linked to the output layer. Batch normalization was applied immediately after each max-pooling layer to enhance stability. The selection of input genes adhered to the following criteria:

- For an input gene, the percentage of cells that this gene experiences dropout must be less than or equal to a threshold of P% (default: 50%).
- The input genes rank in the top five based on expression correlation, evaluated by the Pearson correlation coefficient, with the target genes in the subset. These genes should not be part of the target gene subset. Duplicate genes were removed subsequently.

We partitioned the data into a training set (90%) and a validation set (10%). The model hyperparameters were configured as follows: learning rate = 0.0001, epochs = 150, batch size = 32. Training is terminated if the validation loss does not improve for five consecutive epochs or reaches a maximum of 150 epochs. The early termination mechanism was anchored in the test data’s loss. Mean Squared Error (MSE) served as the chosen loss metric for this study. The loss was computed exclusively between actual expression values and predicted values. The missing expression values of the target gene were masked during the model training and testing processes. After the model was established, the predicted expression levels of these masked data points represent the recovered gene expression values.

### Accuracy evaluation

Given a dataset, we selected genes that were expressed at half or more cells. Cells that had zero expression values for all selected genes were excluded from further analysis. We then randomly chose the dropout points based on the dropout probability of 10%. The expression values in these chosen points were masked out. The predicted expression and true expression of the masked data points were compared for evaluation. Moreover, four additional random datasets were produced by using different random seeds for testing performance robustness.

We employed the mean squared error (MSE) and Pearson correlation coefficient (PCC) for performance assessment and comparison. The calculations for MSE and PCC are as follows:

$$\begin{aligned} MSE= & {} \frac{\sum _{i=1}^{M}(y_{i}-x_{i})^2}{M} \\ PCC= & {} \frac{{}\sum _{i=1}^{M} (x_i - \overline{x})(y_i - \overline{y})}{\sqrt{\sum _{i=1}^{M} (x_i - \overline{x})^2(y_i - \overline{y})^2}} \end{aligned}$$

*M* represents the total number of predicted expression values. $x_{i}$ represents the real expression value, and $y_{i}$ represents the imputed expression value. $\bar{x}$ and $\bar{y}$ denote the real and predicted mean expression levels.

In the RNA FISH validation, we obtained single-cell RNA-seq (scRNAseq) data and its RNA FISH data from a melanoma cell line in the GEO dataset (GSE99330). The scRNAseq dataset consists of 17,511 genes and 4,701 cells, with 92.69% of the data points containing zero values. On the other hand, the RNA FISH dataset comprises 26 genes and 88,040 samples. We focused on a specific cell type identified through the ADPclust clustering method. To align with DeepImpute, we retained five genes with a variance-to-mean ratio greater than 0.5 and also present in both the RNA FISH and scRNAseq datasets. We then normalized the cells using the housekeeping gene *GAPDH* (Glyceraldehyde 3-phosphate dehydrogenase) and subsequently removed outlier cells with extreme *GAPDH* expression (cells below or equal to the 20% and above or equal to the 80%). Next, we rescaled each expression value using a GAPDH-based factor as described below.

$$\begin{aligned} \hat{G_{i,j}} = G_{i,j}\frac{(\sum _{j=1}^{N}G_{GAPDH,j})/N}{{G_{GAPDH,j}}} \end{aligned}$$

Here, $G_{i,j}$ represents the expression of the *i*-th gene in the *j*-th cell, while $\hat{G_{i,j}}$ represents the rescaled value. $G_{\textit{GAPDH},j}$ stands for the expression of the *GAPDH* gene in the *j*-th cell. *N* represents the total number of cells.

Subsequently, we calculated GINI coefficients, Mean Squared Error (MSE), and Pearson Correlation Coefficient (PCC) for each method’s GINI values, following a procedure similar to DeepImpute. The Gini index has the advantage of summarizing the inequality of the entire distribution using a single statistic that ranges from 0 to 1^38^. A lower GINI coefficient reflects a higher degree of equality in this gene. It was calculated using the following equation:

$$\begin{aligned} GINI = \frac{\sum _{i=1}^{N}\sum _{j=1}^{N}|x_{i}-x_{j}|}{2N^2\overline{x}} \end{aligned}$$

Here, N represents the total cell count. The symbol $\overline{x}$ denotes the average value of the entire gene expression vector. $x_{i}$ signifies the gene expression level in the *i*-th cell.

### Cell clusters assessment

The cell clusters To evaluate the alignment between the cell clusters and the true cell types, we employed three metrics: purity, entropy, and the Adjusted Rand Index (ARI). These metrics were used to measure the accuracy and consistency of the cell clustering results.

Entropy and purity were calculated using the R package IntNMF to assess the quality of cell clustering results following the imputation process. The formulas used to compute entropy and purity are based on the following equations, considering l as the total number of cell types and k as the total number of cell clusters.

The entropy of the cell clusters with respect to the known cell types is given by

$$\begin{aligned} Entropy = - \frac{1}{Nlog_{2}l} \sum _{i}^{k} \sum _j^{l} n_{i}^{j} log_{2}( \frac{n_{i}^{j}}{n_{i}}) \end{aligned}$$

Here, N refers to the total number of cells. $n_{i}$ refers to the number of cells in the ith cluster. $n_{i}^{j}$ refers to the number of cells in the *ith* cluster that belongs to the *jth* cell types. A smaller value of entropy indicates that the clusters are more homogeneous

$$\begin{aligned} Purity = \frac{\sum _i ^k max_{j}(c_{i}\cap t_{j})}{N} \end{aligned}$$

Here, N refers to the total number of cells. k is the number of cell clusters. $c_{i}$ denotes ith cluster, while $t_{j}$ denotes the *jth* cell type. Purity is a measure that indicates the overall percentage of cells correctly classified within the cell clusters^39^. The purity value ranges from 0 to 1, where a higher purity value signifies better clustering results.

We also utilized the ARI to assess the clustering results. ARI is a widely used measure for comparing clusters, as it calculates a similarity measure between two clusterings by considering all pairs of samples and counting the pairs that are assigned to the same or different clusters in both the predicted and true clusterings. The ARI was computed as following.

$$\begin{aligned} ARI = \frac{\text {RI} - \mathbb {E}[\text {RI}]}{\max (\text {RI}) - \mathbb {E}[\text {RI}]} \end{aligned}$$

where

$$\begin{aligned} \text {RI}&= \frac{2(\text {TP} \cdot \text {TN} - \text {FP} \cdot \text {FN})}{(\text {TP} + \text {FN})(\text {FN} + \text {TN}) + (\text {TP} + \text {FP})(\text {FP} + \text {TN})} \\ \mathbb {E}[\text {RI}]&= \frac{(\text {TP} + \text {FN})(\text {FN} + \text {TN}) + (\text {TP} + \text {FP})(\text {FP} + \text {TN})}{(\text {TP} + \text {FP} + \text {FN} + \text {TN})^2} \end{aligned}$$

Here, TP refers to true positives, TN refers true negatives, FP stands for false positives, FN stands for false negatives. A higher ARI value indicates a better agreement between the two clusterings.

For cell clustering visualization, we employed t-NSE, which is a non-linear dimensionality reduction technique that transforms high-dimensional data into lower dimensions, allowing for effective visualization of the cell clusters.

### Differential expression analysis

We used the Python package Scanpy^40^ for the differential expression (DE) analysis. We performed pairwise differential analysis for the ESC-DEC dataset, comprising H1 ESC and DEC cell types. Differentially expressed genes (DEGs) were identified at a significance level of $p \le 0.05$, and the resulting DEGs were ranked in descending order of absolute log-fold change (logFC). Simultaneously, in the bulk data, DEGs were obtained using $p \le 0.09$, and the resulting genes were ranked based on the absolute logFC in descending order. For the simulated data, we employed a multi-cell DE analysis approach. Genes with an adjusted p-value of less than or equal to 0.05 were retained and then ranked based on the absolute logFC in descending order. We used the AUC to assess the concordance between the top-ranked DEGs in both bulk and scRNAseq datasets. Utilizing the Pearson correlation coefficient, we measured the correlation of fold changes for common genes between bulk and scRNAseq data. Moreover, we assessed the similarity of fold change for these common genes using the MSE, with bulk-level fold changes as a reference, and the Normalized Euclidean Distance (NED). The NED was computed using the equation below.

$$\begin{aligned} \text {NED} = 0.5\frac{\text {var}(X - Y)}{\text {var}(X) + \text {var}(Y)} \end{aligned}$$

### Datasets used in the assessment

The Jurkat scRNAseq dataset was generated from human Jurkat cell lines using a droplet-based GemCode platform, which allows 3’mRNA counting of tens of thousands of single cells per sample^5^. It was downloaded from 10x Genomics. The Jurkat dataset contained 32,738 genes and 3,258 cells, and after filtering out the genes with zero expression in all cells, 17,753 genes remained, representing 81.99% zero values.

The Grun data^36^ was downloaded from GEO database (GSE54695), it is a dataset that generated from J1 mouse embryonic stem cells (mESCs), which were cultured in 2i or in serum medium. It contains 12,535 genes and 320 cells. After removing genes with zero expression levels in all cells, 52.12% of the remaining expression values were zero.

We acquired FISH data (GSE99330)^41^ from a melanoma cell line, which includes both the Drop-seq data and its RNA FISH dataset. The single-cell RNA-seq data consists of 17,511 genes and 4,701 cells, with 92.69% of the data points representing zero values. On the other hand, the RNA FISH dataset comprises 26 genes and 88,040 samples.

The mouse aortic leukocyte dataset was downloaded from PangelaodDB (SRS2747908)^42^. It comprises 19,150 genes and 377 cells, with 91.23% of the data points being zeros. After removing 513 genes that showed no expression across all cells, the dataset was reduced to 18,637 genes, resulting in a sparsity of 91%. The dataset includes eight cell types with the following cell counts: 87 B cells, 70 T memory cells type 1, 62 Macrophages, 56 T memory cells type 2, 44 Nuocytes, 23 T cells, 19 Neutrophils, and 16 T memory cells type 3.

The Zeisel dataset, accessible through GEO accession number GSE60361, comprises a total of 19,972 genes and 3,005 cells, with 81.21% of the values being zeros. This dataset includes seven cell types with the following cell counts: 224 astrocytes ependymal, 235 endothelial mural, 290 interneurons, 98 microglia, 820 oligodendrocytes, 939 pyramidal CA1, and 399 pyramidal SS.

The paired bulk and single-cell RNAseq of human embryonic stem cells (ESC) and definitive endoderm cells (DEC) datasets were downloaded from GEO (GSE75748). The scRNAseq consists of 19,097 genes and 350 cells, with 212 cells belonging to the H1 ESC type and 138 cells belonging to the DEC type. The dataset contains a total of 49.13% of zero values. On the other hand, the bulk data contains 19,097 genes and 6 samples, with 14.78% zero values.

We utilized the Splatter package^43^ in R to generate simulated scRNAseq data. Splatter enables the simulation of scRNA-seq data based on a gamma-Poisson distribution. It provides users with the flexibility to define cell groups, set probabilities for differentially expressed genes, and simulate dropout events. The package also offers functions for comparing real and simulated datasets. In our simulation, we generated data for 2,000 cells with expression levels for 4,000 genes. The cells were divided into three groups with probabilities of 0.1, 0.1, 0.2, 0.2, and 0.4, respectively. We set the parameters dropout.shape and dropout.mid (representing the midpoint parameter for the dropout logistic function) to -0.5 and 1, respectively.

### Supplementary Information

Supplementary Information.

## Data Availability

The Jurkat scRNAseq dataset is available at 10x Genomics website. The Grun dataset was downloaded from the GEO database with accession number GSE54695. The RNA FISH and its scRNAseq dataset are available on the GEO database with accession number GSE99330. The mouse aortic leukocyte dataset is accessible on PangelaodDB with accession number SRS2747908 (PangelaoDB). The Zeisel dataset can be found on the GEO database with accession number GSE60361. The paired bulk and single-cell RNAseq data of human embryonic stem cells (ESC) and definitive endoderm cells are available on the GEO database with accession number GSE75748.
