# Supplementary material for: cnnImpute: missing value recovery for single cell RNA sequencing data
Source: Sci Rep. 2024 Feb 16;14:3946. doi: 10.1038/s41598-024-53998-x (PMC10873334; doi:10.1038/s41598-024-53998-x)
Supplement: Supplementary file 1 — Supplementary Information. [file 41598_2024_53998_MOESM1_ESM.pdf]

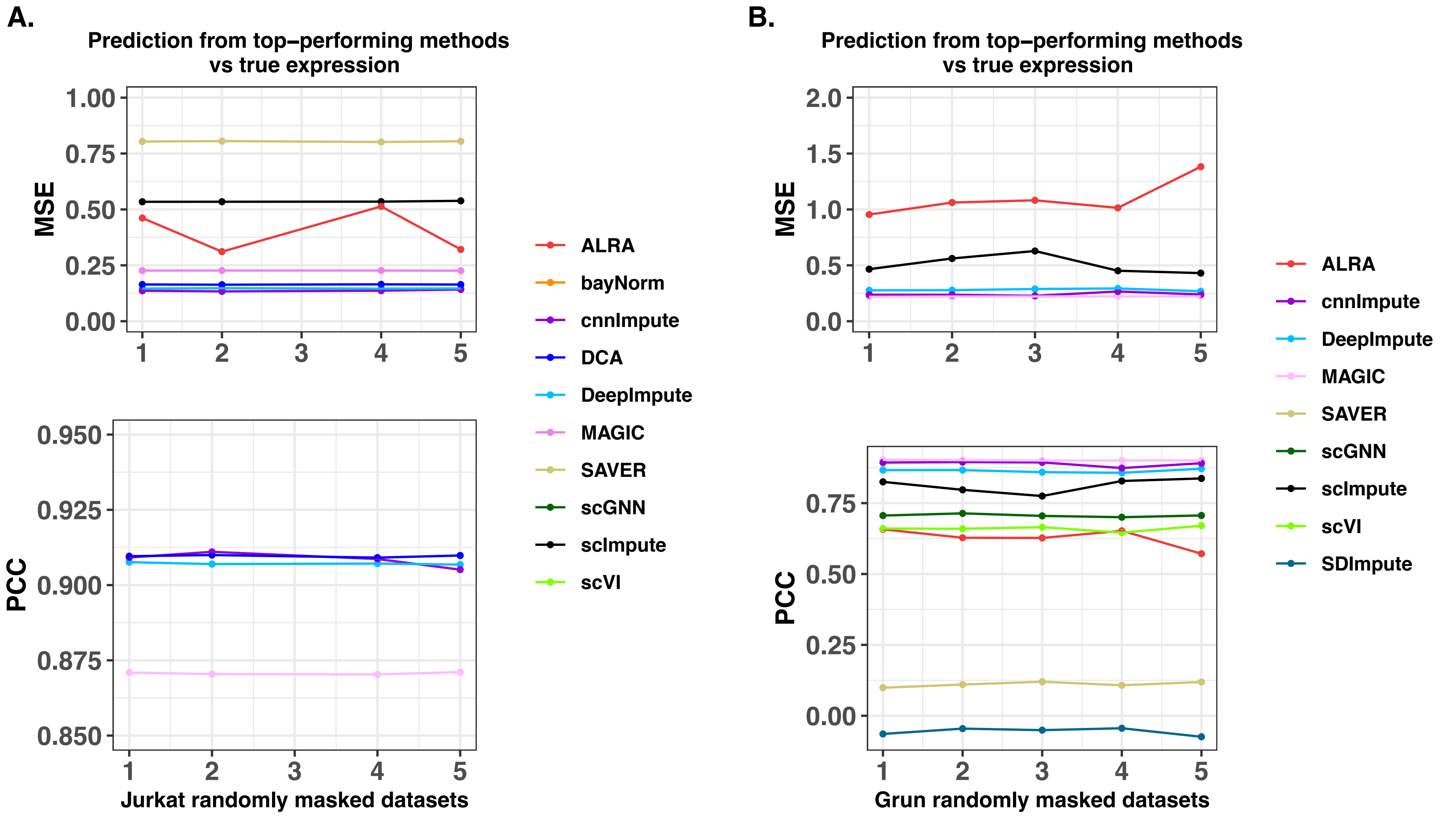

Supp. Figure 1. The mean square error (MSE) and Pearson correlation coefficient (PCC) of top−ranked methods.

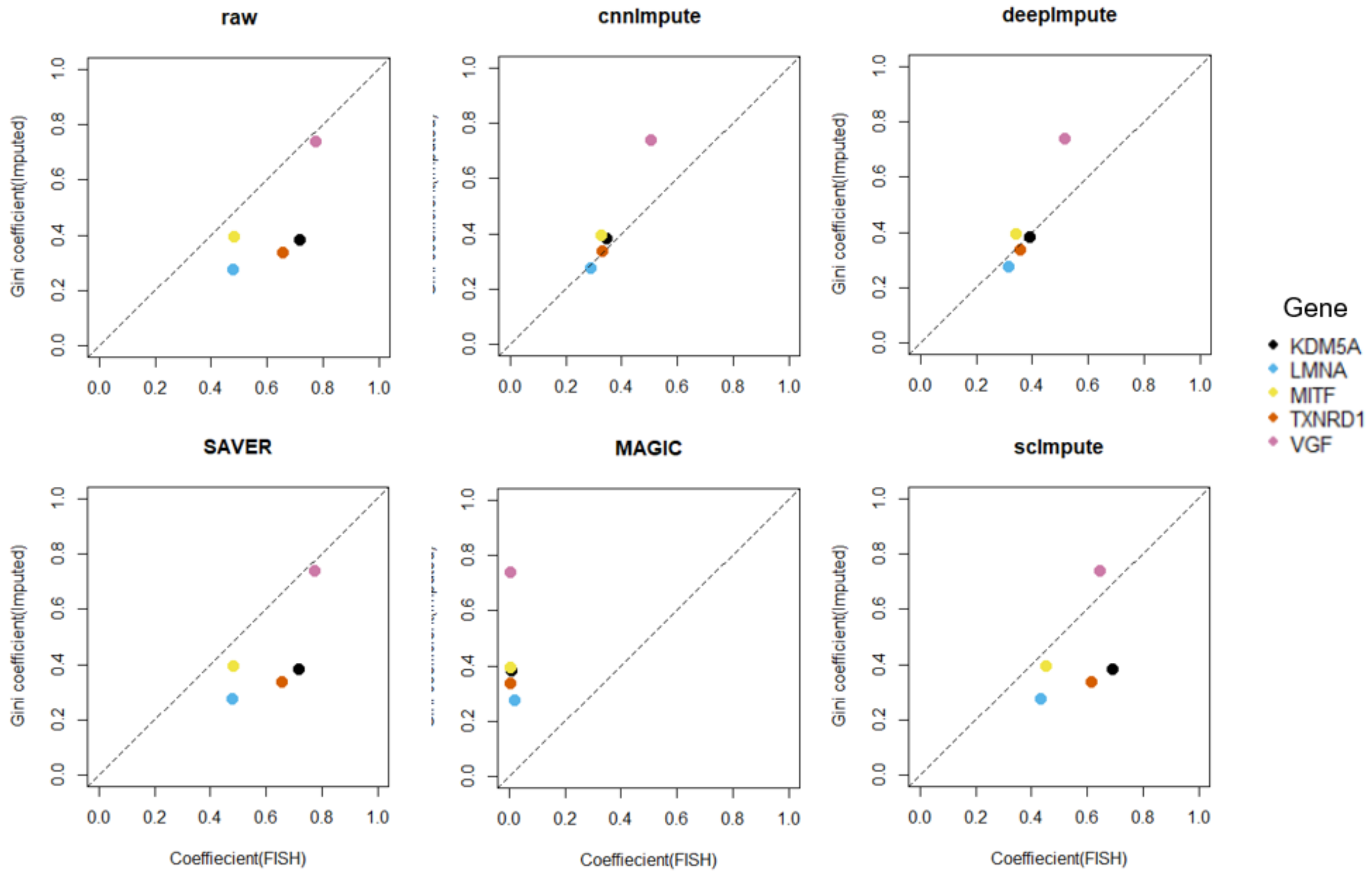

Supp. Figure 2. The correlation of Gini coefficients from FISH RNA and scRNA-seq

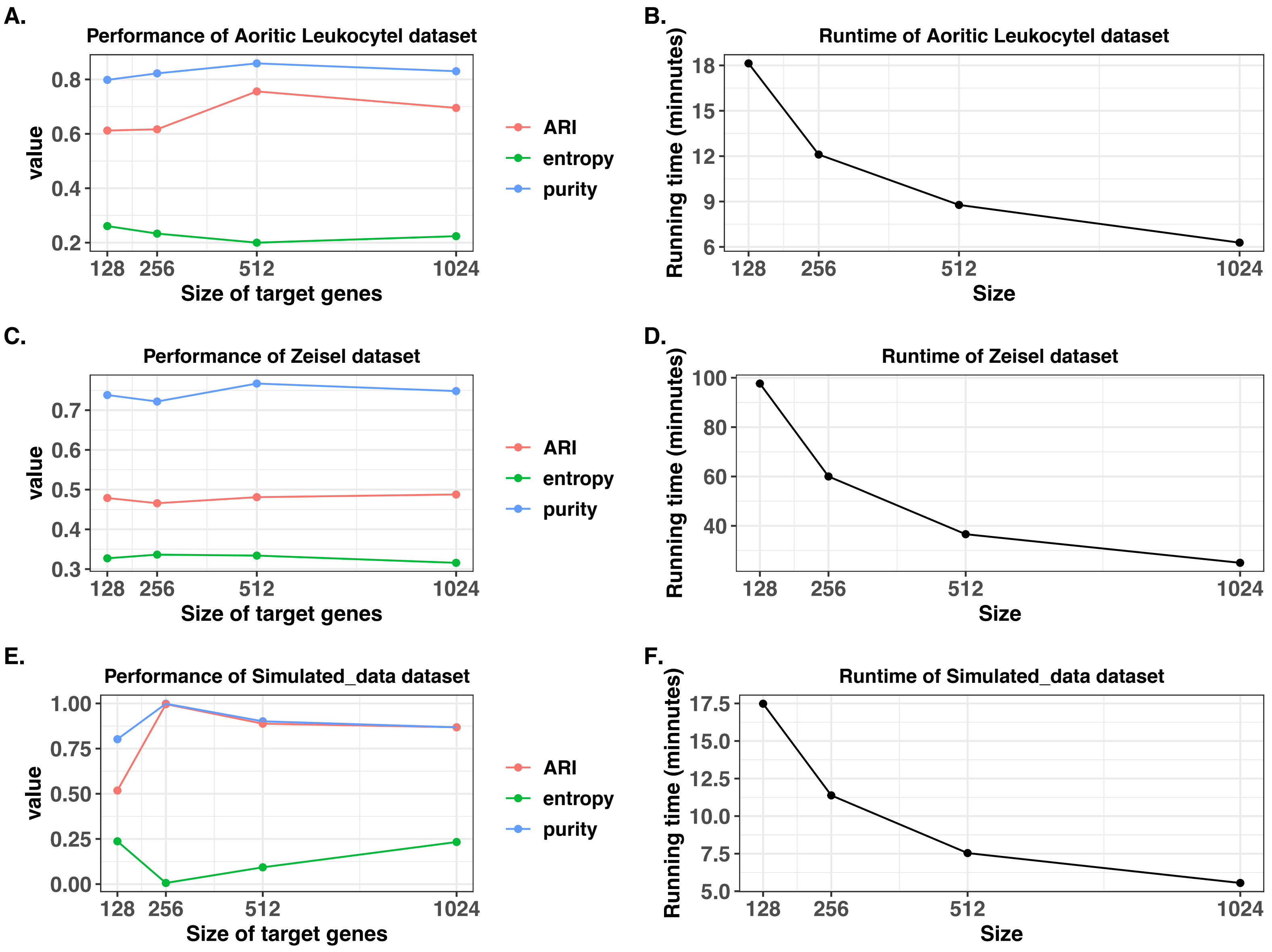

Supp. Figure 3. The cnnImpute performance vs the size of target genes.

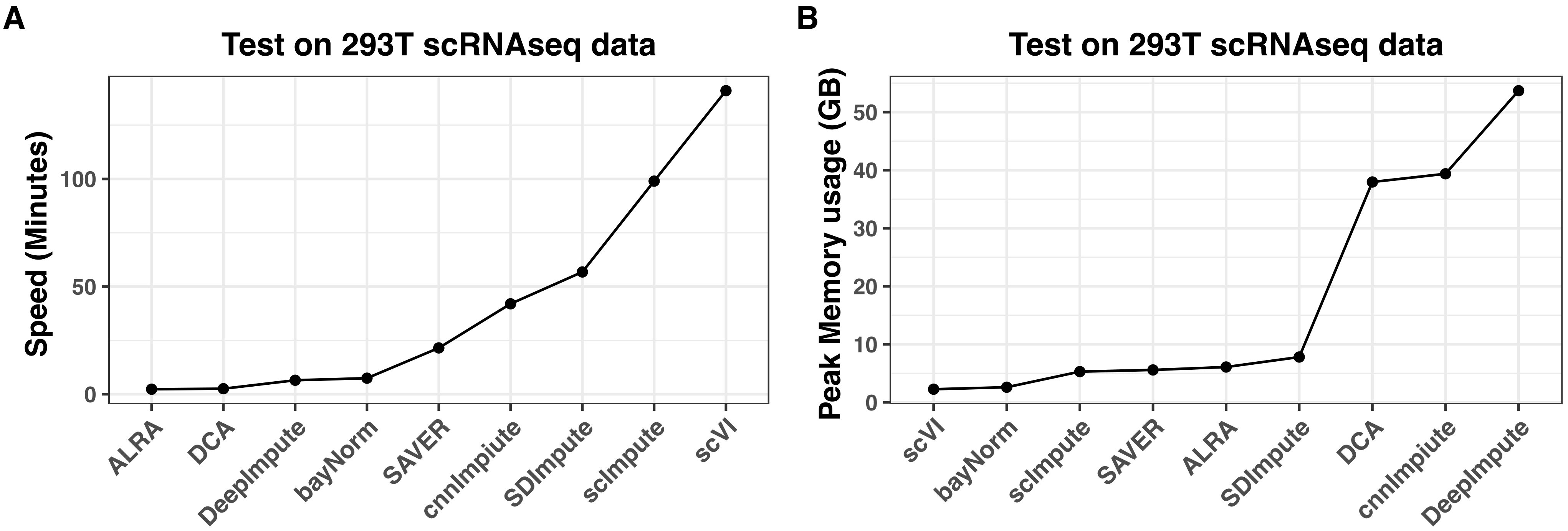

Supp. Figure 4. The running speed and memory usage of different methods.
